# Recovery Pattern of High-Frequency Acceleration Vestibulo-Ocular Reflex in Unilateral Vestibular Neuritis: A Preliminary Study

**DOI:** 10.3389/fneur.2019.00085

**Published:** 2019-03-07

**Authors:** Wei Fu, Feng He, Dong Wei, Ya Bai, Ying Shi, Xiaoming Wang, Junliang Han

**Affiliations:** ^1^Department of Geriatrics, Xijing Hospital, Fourth Military Medical University, Xi'an, China; ^2^Department of Neurology, Xijing Hospital, Fourth Military Medical University, Xi'an, China

**Keywords:** video head impulse test, saccades, covert saccades, vestibulo-ocular reflex, vestibular compensate, vestibular function, horizontal semicircular canal

## Abstract

**Objective:** To explore the recovery pattern of the high-frequency acceleration vestibulo-ocular reflex (VOR) function in unilateral vestibular neuritis (UVN).

**Methods:** Forty-seven consecutive patients with UVN were recruited within 10 days of symptom onset for this study. The high-frequency acceleration horizontal VOR function was assessed using the video head impulse test (vHIT). Patients returned for follow-up evaluation at ~6 months after the onset of symptoms. According to the dizziness handicap inventory questionnaire (DHI), the patients were classified into the normal to mild dizziness group (DHI score ≤30) and moderate to severe dizziness group (DHI score >30) at the follow-up. All the obtained horizontal vHIT gains and corrective saccades parameters were analyzed.

**Results:** vHIT results showed a significantly horizontal VOR gain recovery in UVN patients at the follow-up on the lesion side (*p* < 0.01). A significantly reduction in the occurrence of corrective saccades (overt and covert) and velocity of corrective saccades (overt and covert) were observed at the follow-up (*p* < 0.05). At the follow-up, the normal to mild dizziness group (DHI score ≤30) had a significantly higher normal rate of VOR gain, the mean vHIT gains and occurrence of isolated covert saccades (*P* < 0.05). Furthermore, the occurrence of mixed saccades and the mean velocity of covert saccades were significantly lower in normal to mild dizziness group (*P* < 0.05).

**Conclusion:** Apart from the recovery of the VOR gain, recovery pattern of corrective saccades can play a key role in vestibular compensate.

## Introduction

Vestibular neuritis (VN) is a common disease causing acute attacks of vertigo. It is characterized by sudden onset of prolonged vertigo with unidirectional spontaneous horizontal–torsional nystagmus, absence of other auditory, or neurologic findings (1). And the vestibulo-ocular reflex (VOR) hypofunction of VN can be identified by caloric irrigation test and head impulse test (HIT) (2). HIT is a simple and valid means of evaluating VOR function in the high-frequency range of 4–7 Hz (3). The subject maintains fixation on an static target while the operator applied unpredictable, sudden, passive, head turns in the horizontal plane, and examiner looks for corrective saccades (4). If the VOR function is intact, the subject can stabilize their gaze on a target during head rotations. However, if the VOR function is deficient, the eyes fail to fix on the target and the patient makes a corrective saccades to refixate the target (4).There are two corrective saccades types: overt saccades and covert saccades. HIT can detect overt saccades, but covert saccades is invisible (5). Fortunately, the video head impulse test (vHIT) can recognize covert saccades and quantifying the VOR gain (eye velocity/head velocity) (6). Thus, vHIT provides a tool that allowing analysis of VOR gain and the corrective saccades, seems to offer new interesting perspectives.

In addition, vHIT is also a quantitative baseline to track recovery of VOR dysfunction. In this study, we measured the horizontal VOR gain and corrective saccades in unilateral vestibular neuritis (UVN) patients from the acute stage to the follow-up. And we also analyzed the correlation with parameters of vHIT and chronic symptoms (dizziness handicap inventory questionnaire) at the follow-up in order to determine which parameters of vHIT are better to predict symptom recovery.

## Methods

### Participants

We identified 47 consecutive patients with acute UVN from the period between 2016 and 2018 (25 men, 22 women; mean age 56.8 years; ranging from 19 to 71 years). The diagnostic criteria for UVN included the following: a history of sudden vertigo (more than 24 h) with unidirectional spontaneous horizontal–torsional nystagmus, and caloric examine showing a lack of unilateral caloric response (canal paresis >25%) (1). Exclusion criteria: (1) History of neurologic disorders or auditory disorders; (2) patients with tumors, traumatic and infection; (3) magnetic resonance imaging (MRI) which revealed brain lesions. All acute UVN patients were treated immediately with corticosteroids (tapering over 2 weeks) and vitamin B12.

The 47 UVN patients were initially examined using vHIT at the acute phase (within 10 days) after onset of vertigo and then scheduled for follow-up examination ~6 months later. In addition, all patients also were inquired the recovery condition of symptom and completed the chinese version of the dizziness handicap inventory (DHI) questionnaire at the follow-up (7). And the patients were classified into the normal to mild dizziness group (DHI score ≤30) and moderate to severe dizziness group (DHI score >30) at the follow-up.

All subjects provided written informed consents to participate in this study. The study was approved by the Institutional Review Board of Xijing Hospital, Fourth Military Medical University.

### vHIT

The vHIT was recorded in all subjects using the ICS Impulse system (Otometrics, Denmark). The horizontal canals were evaluated. The subject was instructed to gaze at a target that was 1.2 m away. First, calibration is performed. In each trial, the examiner stood behind the patients and performed head impulses by a small angle (~10–20°) and an appropriate velocity (150–200°/s). And 20 impulses were recorded for each direction. In our study, the horizontal vHIT were performed with jaw hand position (8). The mean horizontal vHIT gains and corrective saccades parameters (covert and overt) were measured. Abnormal criteria is the horizontal vHIT gain values <0.8 and corrective saccades peak velocity >100°/s (9, 10).

### Statistical Analysis

Statistical comparisons of the horizontal vHIT gains, peak head velocities, and corrective saccades parameters (velocity of saccade and latency of saccade) at the acute stage and the follow-up of UVN were made using the Student's *t*-test, and occurrence of saccades (overt and covert) at the acute stage and the follow-up were compared with Chi-square test or Fisher exact test. A Student's *t*-test, Chi-square test, and Fisher exact test also were used to investigate the relationship between two DHI groups (DHI score ≤30 and DHI score >30) and vHIT results at the follow-up. Spearman's correlation test was used to analyze the correlation between the DHI score and vHIT gains or corrective saccades parameters. *p* < 0.05 was considered statistically significant. Statistical analysis were done by using SPSS software (version19, SPSS Inc., Chicago, IL, USA).

## Results

In 47 UVN patients, the mean peak head velocity on the lesion side was 174.44 ± 18.32°/s at the acute stage and 175.19 ± 17.49°/s at the follow-up, respectively. The mean peak head velocity on the healthy side was 175.14 ± 16.10°/s at the acute stage and 174.14 ± 15.85°/s at the follow-up, respectively. There was no significant difference in the mean peak head velocity (lesion side and healthy side) between the acute stage and the follow-up (*p* > 0.05, Table 1).

**Table 1:** vHIT results in unilateral vestibular neuritis patients at the acute stage and the follow-up examination (*n* = 47).

	**Acute stage**	**Follow-up**	***p*-value**
**PEAK HEAD VELOCITIES**			
Lesion-side mean head velocity (°/s)	174.44 ± 18.32	175.19 ± 17.49	>0.05^a^
Healthy-side mean head velocity (°/s)	175.14 ± 16.10	174.14 ± 15.85	>0.05^a^
**vHIT Gain**			
Lesion-side mean gain	0.47 ± 0.15	0.69 ± 0.23	< 0.01^a^
Healthy-side mean gain	0.97 ± 0.12	1.00 ± 0.13	>0.05^a^
**OVERT SACCADE PARAMETERS**			
Occurrence of overt saccade (%)	100(47/47)	59.58(28/47)	< 0.01^b^
Velocity of overt saccade (°/s)	203.00 ± 62.00	152.46 ± 29.70	< 0.01^a^
Latency of overt saccade (ms)	310.02 ± 41.91	314.21 ± 38.84	>0.05^a^
**COVERT SACCADE PARAMETERS**			
Occurrence of covert saccade (%)	100(47/47)	87.23(41/47)	<0.05^c^
Velocity of covert saccade (°/s)	209.23 ± 48.17	186.14 ± 45.69	<0.05^a^
Latency of covert saccade (ms)	130.30 ± 24.91	123.41 ± 24.47	>0.05^a^

a *t-test*.

b *Chi-square test*.

c *Fisher exact test*.

### vHIT Parameters at the Acute Stage and the Follow-Up

The mean horizontal vHIT gains on the lesion side were 0.47 ± 0.15 at the acute stage and 0.69 ± 0.23 at the follow-up, respectively, and the gains at the follow-up were significantly higher than the gains at the acute stage (*p* < 0.01, Table 1). The healthy side had the mean horizontal vHIT gains were 0.97 ± 0.12 and 1.00 ± 0.13 at the acute stage and the follow-up, respectively, and this was not significantly different (*p* > 0.05, Table 1). The occurrence of overt saccade were 100% at the acute stage and 59.58% at the follow-up, respectively, and the occurrence of overt saccade showed a significant decrease at the follow-up (*p* < 0.01, Table 1). The occurrence of covert saccade were 100% at the acute stage and 87.23% at the follow-up, respectively, and the occurrence of covert saccade showed a significant decrease at the follow-up (*p* < 0.05, Table 1). The mean velocity of overt saccades were 203.00 ± 62.00°/s at the acute stage and 152.46 ± 29.70°/s at the follow-up, respectively, and the mean velocity of overt saccades showed a significant decrease at the follow-up (*p* < 0.01, Table 1). The mean velocity of covert saccades were 209.23 ± 48.17°/s at the acute stage and 186.14 ± 45.69°/s at the follow-up, respectively, and the mean velocity of covert saccades also decreased significantly at the follow-up (*p* < 0.05, Table 1). The mean latency of overt saccades were 310.02 ± 41.91 ms at the acute stage and 314.21 ± 38.84 ms at the follow-up, respectively, and this was not significantly different (*p* > 0.05, Table 1). The mean latency of covert saccades were 130.30 ± 24.91 ms at the acute stage and 123.41 ± 24.47 ms at the follow-up, respectively, and this also was not significantly different (*p* > 0.05, Table 1).

### vHIT Parameters and Symptoms in UVN Patients at the Follow-Up

**Figure 1:**
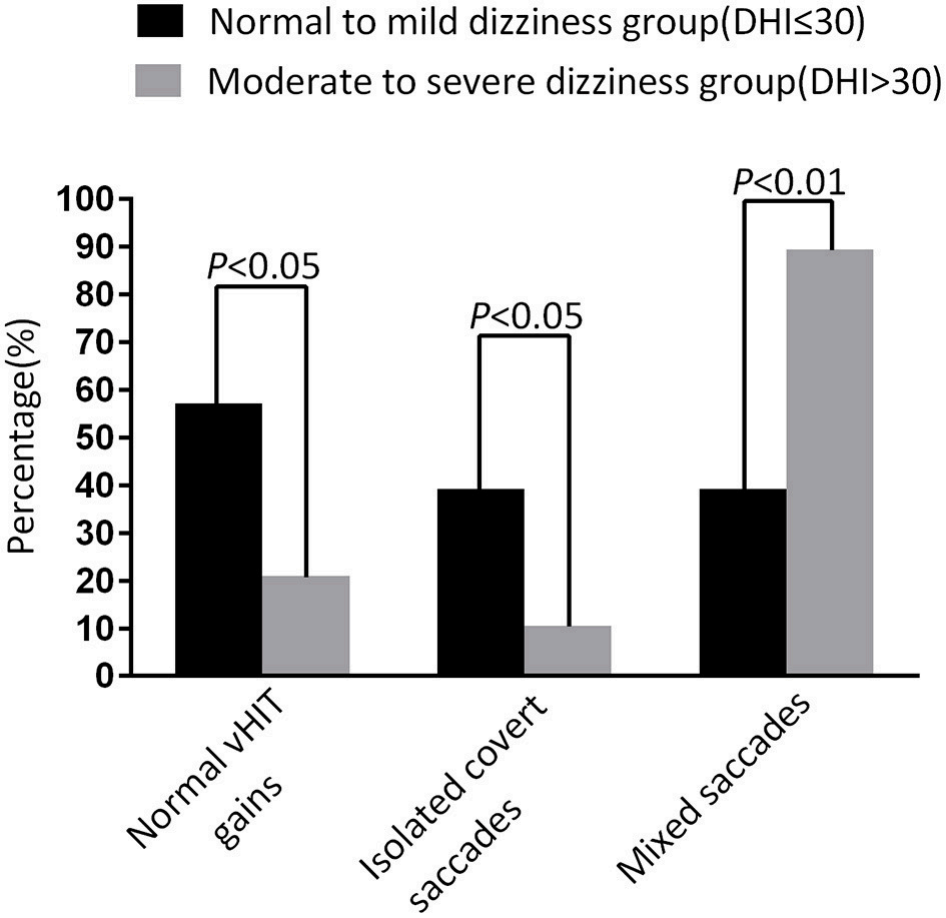
Comparison of normal vHIT gains rate, occurrence of isolated covert saccades, and occurrence of mixed saccades measured from normal to mild dizziness group (DHI ≤30) and moderate to severe dizziness group (DHI >30) with UVN at the follow-up. The normal to mild dizziness group (DHI ≤30) were significantly more likely to have a normal vHIT gain, high occurrence of isolated covert saccades, and low occurrence of mixed saccades.

**Figure 2:**
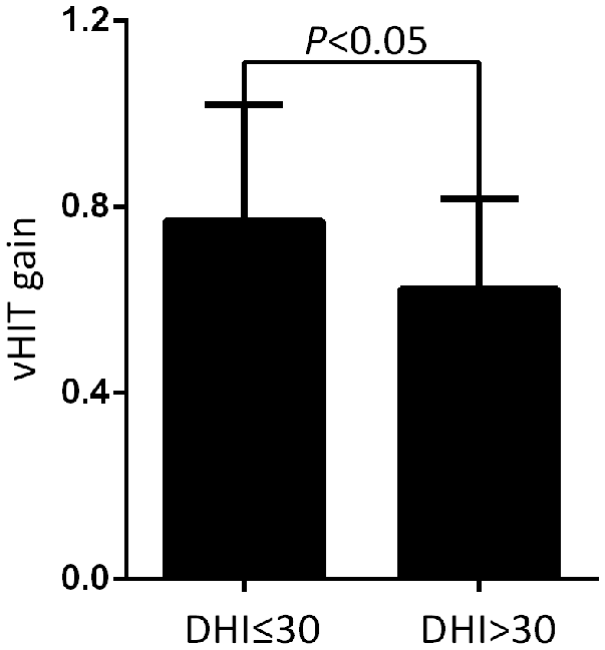
Comparison of the mean horizontal vHIT gains on the lesion side from normal to mild dizziness group (DHI ≤30) and moderate to severe dizziness group (DHI >30) with UVN at the follow-up. The mean gains in the normal to mild dizziness group (DHI ≤30) were significantly higher than the moderate to severe dizziness group (DHI >30).

**Figure 3:**
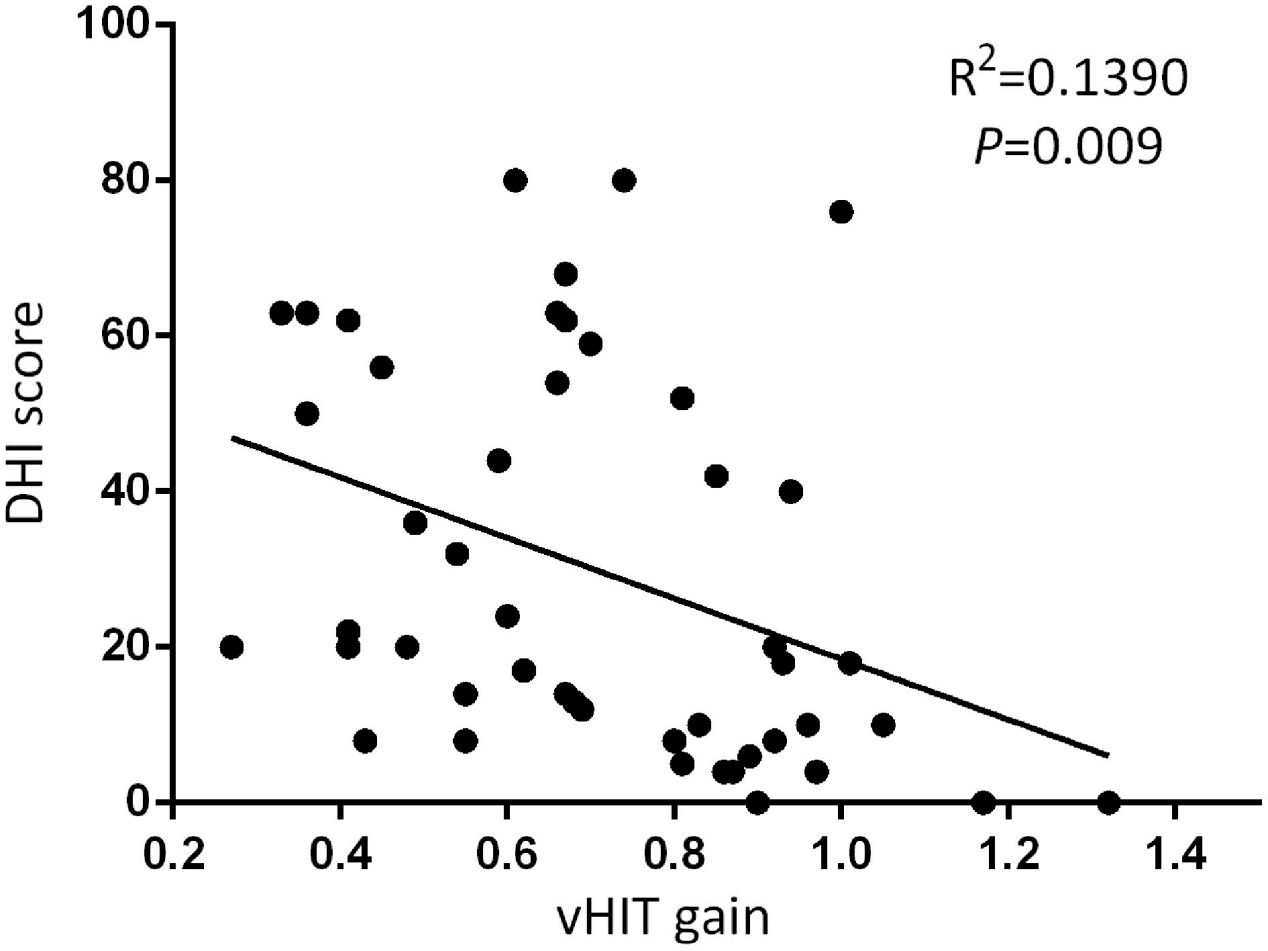
The DHI score was negatively correlated with lesion side vHIT gains at the follow-up.

**Figure 4:**
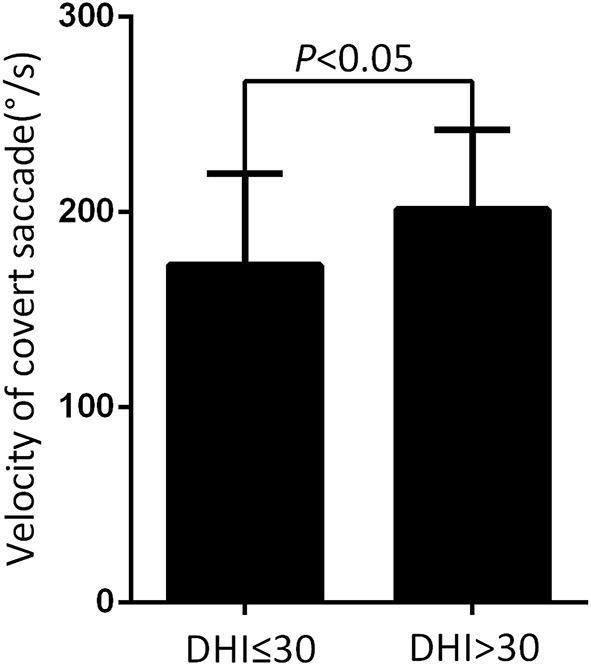
Comparison of the mean velocity of covert saccade from normal to mild dizziness group (DHI ≤30) and moderate to severe dizziness group (DHI >30) with UVN at the follow-up. The mean velocity of covert saccades at the normal to mild dizziness group (DHI ≤30) were significantly lower than the moderate to severe dizziness group (DHI >30).

**Figure 5:**
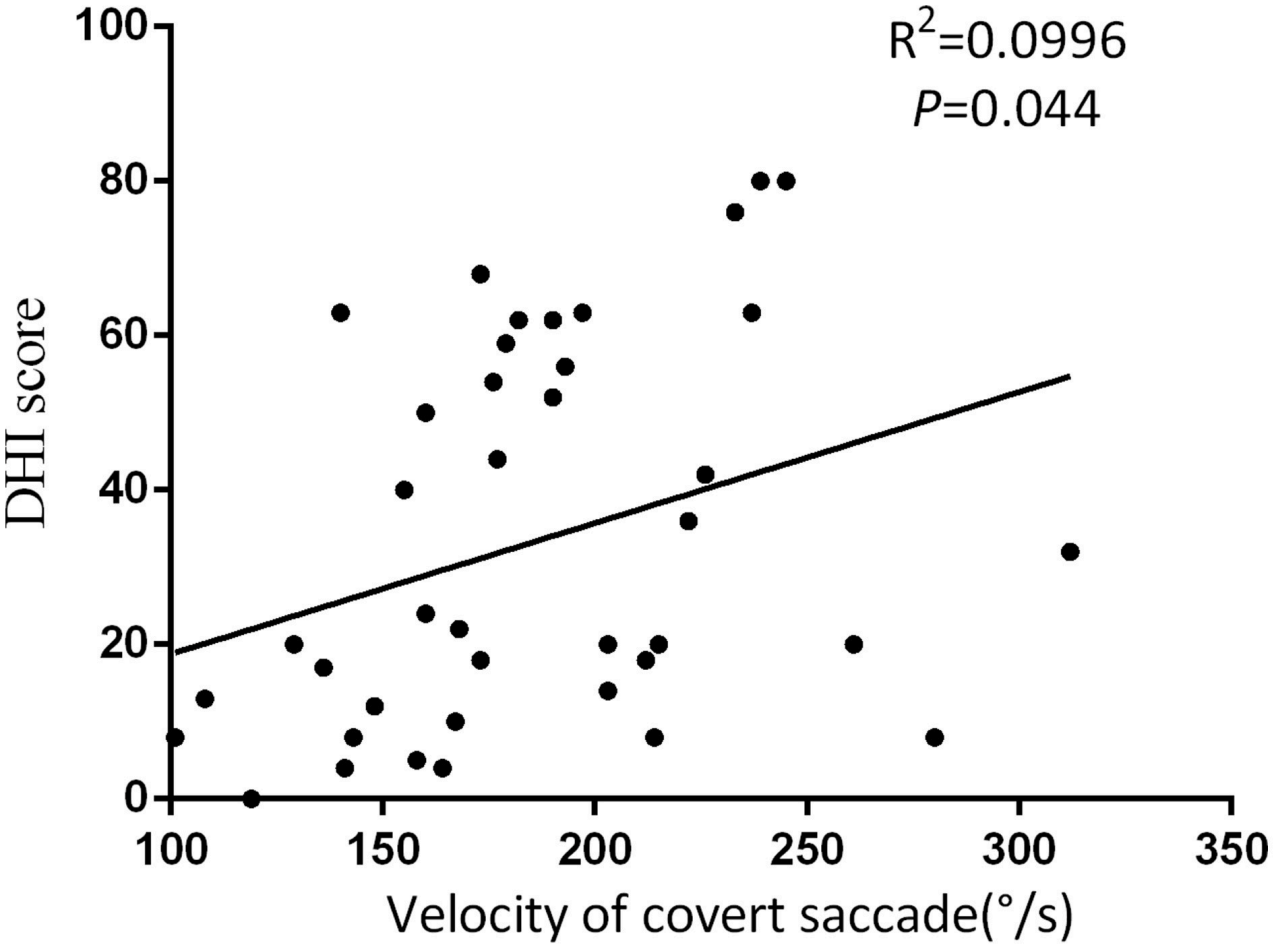
The DHI score was positively correlated with velocity of covert saccades at the follow-up.

At the follow-up, the DHI score was equal to or <30 (normal to mild dizziness group) in 28 UVN patients and >30 (moderate to severe dizziness group) in 19 UVN patients. 57.14% UVN patients in the normal to mild dizziness group and 21.05% UVN patients in the moderate to severe dizziness group had a normal vHIT gains at the follow-up. And the proportion of patients with normal vHIT gains were significantly higher in the normal to mild dizziness group (*p* < 0.05, Table 2, Figure 1). The mean horizontal vHIT gains on the lesion side were 0.77 ± 0.25 in the normal to mild dizziness group and 0.62 ± 0.19 in the moderate to severe dizziness group, respectively, and the mean gains in the normal to mild dizziness group were significantly higher than the moderate to severe dizziness group (*p* < 0.05, Table 2, Figure 2). The DHI score was negatively correlated with lesion side vHIT gains (*R* = −0.372, *p* = 0.009, Figure 3). 21.42% UVN patients in the normal to mild dizziness group and 0% UVN patients in the moderate to severe dizziness group had not a corrective saccades at the follow-up, and this was not significantly different (*p* = 0.06, Table 2). The proportion of patients with occurrence of isolated covert saccades in the normal to mild dizziness group (39.29%) were significantly higher than the moderate to severe dizziness group (10.53%) (*p* < 0.05, Table 2, Figure 1). However, the proportion of patients with occurrence of mixed saccades (overt and covert) in the normal to mild dizziness group (39.29%) were significantly lower than the moderate to severe dizziness group (89.47%) (*p* < 0.01, Table 2, Figure 1). Furthermore, the mean velocity of overt saccades were 155.10 ± 33.60°/s and 160.52 ± 30.94°/s in normal to mild dizziness group and moderate to severe dizziness group at the follow-up, respectively, and this was not significantly different (*p* > 0.05, Table 2). The mean velocity of covert saccades were 170.41 ± 45.15°/s and 201.10 ± 40.46°/s in normal to mild dizziness group and moderate to severe dizziness group at the follow-up, respectively, and the mean velocity of covert saccades in normal to mild dizziness group were significantly lower than the moderate to severe dizziness group (*p* < 0.05, Table 2, Figure 4). The DHI score was positively correlated with velocity of covert saccades (*R* = 0.315, *p* = 0.044; Figure 5). The mean latency of overt saccades were 308.93 ± 46.84 ms and 306.97 ± 30.96 ms in normal to mild dizziness group and moderate to severe dizziness group at the follow-up, respectively, and this was not significantly different (*p* > 0.05, Table 2). The mean latency of covert saccades were 124.85 ± 27.21 ms and 123.43 ± 22.45 ms in normal to mild dizziness group and moderate to severe dizziness group at the follow-up, respectively, and this also was not significantly different (*p* > 0.05, Table 2).

**Table 2:** vHIT results in unilateral vestibular neuritis patients with normal to mild dizziness and moderate to severe dizziness at the follow-up (*n* = 47).

	**Normal to mild dizziness (DHI ≤30; *n* = 28)**	**Moderate to severe dizziness (DHI > 30; *n* = 19)**	***p*-value**
Normal vHIT gain (%)	57.14(16/28)	21.05(4/19)	<0.05^b^
Lesion-side mean gain	0.77 ± 0.25	0.62 ± 0.19	<0.05^a^
Without saccade (%)	21.42(6/28)	0(0/19)	0.06^c^
Occurrence of isolated covert saccade (%)	39.29(11/28)	10.53(2/19)	<0.05^b^
Occurrence of mixed saccade (%)	39.29(11/28)	89.47(17/19)	<0.01^b^
Velocity of overt saccade (°/s)	155.10 ± 33.60	160.52 ± 30.94	>0.05^a^
Latency of overt saccade (ms)	308.93 ± 46.84	306.97 ± 30.96	>0.05^a^
Velocity of covert saccade (°/s)	170.41 ± 45.15	201.10 ± 40.46	<0.05^a^
Latency of covert saccade (ms)	124.85 ± 27.21	123.43 ± 22.45	>0.05^a^

a *t-test*.

b *Chi-square test*.

c *Fisher exact test. DHI, dizziness handicap inventory*.

## Discussion

VN has long been known to affect the superior vestibular nerve, inferior vestibular nerve, or both (11, 12). The vHIT advent has provided clinicians with diagnostic tools to identify dysfunctions of the pathway of each single semicircular canals. It became evident that both nerve divisions could be affected, both together and independently or rare patterns of the ampullary afferents (13–15).

In recent years, there have been some studies of using vHIT in patients with UVN, but these studies have focused on diagnostic efficacy evaluation and gain is a common parameter to track deterioration or recovery of VOR function at the follow-up (9, 16). Few studies have evaluated quantitatively the corrective saccades in UVN. In our study, we investigated both gain and corrective saccades parameters of the horizontal semicircular canal changes in UVN patients from the acute stage to the follow-up. We found that lesion side vHIT gains always improved to a varying extent at the follow-up. On the healthy side, the vHIT gain was normal even in the acute stage and no worsening of gain could be found at the follow-up. Buki et al. reported that lesion side vHIT gains improved and healthy side vHIT gains were in the normal range at 2 months follow-up in 44 VN patients (16). After testing with search coils in 37 VN patients at different time periods (1–240 weeks), palla et al. revealed the similar result (17). This is consistent with the result obtained by us in VN patients. The gains recovery depended on restoration of the horizontal semicircular canals function. It has some reasons to explain such recovery. Firstly, regeneration of peripheral sensory hair cells. Secondly, sprouting of new afferent terminals from remaining fibers in the vestibular nerve. Thirdly, increased synaptic weight of remaining vestibular inputs (18). These mechanisms include cellular recovery, spontaneous re-establishment of the tonic firing rate centrally (19).

However, vHIT gain is not always recover quickly and abnormal vHIT gain could persists for a long time. Sometimes there is rarely recovery of high-frequency acceleration VOR function in our daily life (20). The major question remains that how compensate slow phase eye velocity in our daily life. Fortunately, there is a saccadic substitution of the slow phase eye movement, thereby preventing dynamic VOR deficit during natural head accelerations (20). However, previous studies focus on the recovery of vHIT gain without sufficient recognition of the fact that changes in corrective saccades parameters are a very effective way of overcoming the inadequate VOR function. Therefore, we evaluated the saccades parameters (overt and covert) of the horizontal semicircular canal in UVN by means of the vHIT at the acute stage and the follow-up. And the corrective saccades was analyzed quantitatively. We observed a significant gradual reduction in the occurrence of saccades (overt and covert) at the follow-up. Interestingly, the decrease of occurrence of overt saccades was significantly more evident at the follow-up (100% at the acute stage vs. 59.58% at the follow-up). However, covert saccade still was detected in most of UVN patients (100% at the acute stage vs. 87.23% at the follow-up). In addition, in our study, velocity of saccade (overt and covert) was also significantly decreased at the follow-up. However, latency of saccade (overt and covert) no significant differences at the acute stage and the follow-up. Martin-Sanz et al. found a significantly faster reduction in the velocity and organization of the compensatory saccades was observed in UVN patients recovery stage (21). Furthermore, a recent study by Yang et al. found that the incidence of corrective saccades and peak velocities of corrective saccades had decreased significantly at the 1-month follow-up (22). Our results were similar to these studies. These results show that saccades and vHIT gain play the same role in the vestibular compensation process.

Due to poor vestibular compensation, some UVN patients have difficulties recovering the VOR (18). They were troubled by chronic dizziness, disequilibrium, and limitations in daily activities (23). It is very important in identifying characteristics of vestibular function tests in good vestibular compensation patients and poor vestibular compensation patients. Kim et al. reported that patients have a positive bedside head impulse test (bHIT) result on follow-up, they are more likely to be dizzy (24). As we know, when performing a bHIT, overt saccades are detectable for the clinician but covert saccades cannot be detected. Due to the lack of quantitative analysis, Kim et al. study only reveal the occurrence of overt saccades can predict symptom recovery. Therefore, one great benefit of vHIT is that it allow the clinician to identify such recovery of peripheral vestibular function. To determine which parameters of HIT are better to predict symptom recovery. In our study, we chose DHI score to quantify symptom during the follow-up. The UVN patients were divided into two groups according to the final DHI score: the normal to mild handicap group (DHI ≤30) and the moderate to severe handicap group (DHI >30). And we found that most of patients have normal vHIT gain and/or isolated covert saccades in the normal to mild group (DHI ≤30). The mean gains at the normal to mild dizziness group were significantly higher than the moderate to severe dizziness group. However, patients with abnormal vHIT gain and/or mixed saccades continue to be troubled by some or all of chronic symptoms. Cerchiai et al. reported that lower values of VOR gain and a high occurrence of overt saccades could give rise to a worse prognosis after acute unilateral vestibulopathy (25). Besides, Wettstein et al. found that it has correlation between compensatory covert saccades and improved performance of dynamic visual acuity-testing in patients with unilateral peripheral vestibular loss (26). These studies results were similarly our research findings. And compare overt saccades with covert saccades, covert saccades can play a very key role in compensation of inadequate VOR response and return to a normal lifestyle (20). In addition, we further found that velocity of covert saccades decline probably related to symptom recovery. The diminution of covert saccades velocity is more obvious in the normal to mild group (DHI ≤30). It may act to minimize the effect of the unilateral vestibular loss on the patient dynamic VOR deficit and conceal their dysfunction (21).

### Limitations of the Study

There are some limitations in this study. First, the number of subjects and follow-up time were insufficient. The future study with a large samples and longer follow-up time need to further confirm our results. Second, we only analyzed the horizontal semicircular canals without analyzing the vertical semicircular canals. This was due to most of VN affected the superior vestibular nerve (27). And horizontal VOR was damage and saccades were more easily observed. Last, we only recruit UVN patients. Further studies are necessary to investigate the recovery pattern in different vestibular diseases, such as bilateral vestibular hypofunction (BVH), meniere's disease (MD), or vestibular migraine (VM).

## Conclusion

Apart from the recovery of the VOR gain, recovery pattern of corrective saccades can play a key role in vestibular compensate.

## Author Contributions

WF designed the experiment, analyzed the data, and wrote the article. FH, DW, YB, and YS collected data and prepared figures. XW and JH guided the study.

### Conflict of Interest Statement

The authors declare that the research was conducted in the absence of any commercial or financial relationships that could be construed as a potential conflict of interest.
